# Prompt Engineering Paradigms for Medical Applications: Scoping Review

**DOI:** 10.2196/60501

**Published:** 2024-09-10

**Authors:** Jamil Zaghir, Marco Naguib, Mina Bjelogrlic, Aurélie Névéol, Xavier Tannier, Christian Lovis

**Affiliations:** 1 Division of Medical Information Sciences Geneva University Hospitals Geneva Switzerland; 2 Department of Radiology and Medical Informatics University of Geneva Geneva Switzerland; 3 Université Paris-Saclay CNRS Laboratoire Interdisciplinaire des Sciences du Numérique Orsay France; 4 Sorbonne Université INSERM Université Sorbonne Paris-Nord, Laboratoire d'Informatique Médicale et d'Ingénierie des Connaissances en eSanté, LIMICS Paris France

**Keywords:** prompt engineering, prompt design, prompt learning, prompt tuning, large language models, LLMs, scoping review, clinical natural language processing, natural language processing, NLP, medical texts, medical application, medical applications, clinical practice, privacy, medicine, computer science, medical informatics

## Abstract

**Background:**

Prompt engineering, focusing on crafting effective prompts to large language models (LLMs), has garnered attention for its capabilities at harnessing the potential of LLMs. This is even more crucial in the medical domain due to its specialized terminology and language technicity. Clinical natural language processing applications must navigate complex language and ensure privacy compliance. Prompt engineering offers a novel approach by designing tailored prompts to guide models in exploiting clinically relevant information from complex medical texts. Despite its promise, the efficacy of prompt engineering in the medical domain remains to be fully explored.

**Objective:**

The aim of the study is to review research efforts and technical approaches in prompt engineering for medical applications as well as provide an overview of opportunities and challenges for clinical practice.

**Methods:**

Databases indexing the fields of medicine, computer science, and medical informatics were queried in order to identify relevant published papers. Since prompt engineering is an emerging field, preprint databases were also considered. Multiple data were extracted, such as the prompt paradigm, the involved LLMs, the languages of the study, the domain of the topic, the baselines, and several learning, design, and architecture strategies specific to prompt engineering. We include studies that apply prompt engineering–based methods to the medical domain, published between 2022 and 2024, and covering multiple prompt paradigms such as prompt learning (PL), prompt tuning (PT), and prompt design (PD).

**Results:**

We included 114 recent prompt engineering studies. Among the 3 prompt paradigms, we have observed that PD is the most prevalent (78 papers). In 12 papers, PD, PL, and PT terms were used interchangeably. While ChatGPT is the most commonly used LLM, we have identified 7 studies using this LLM on a sensitive clinical data set. Chain-of-thought, present in 17 studies, emerges as the most frequent PD technique. While PL and PT papers typically provide a baseline for evaluating prompt-based approaches, 61% (48/78) of the PD studies do not report any nonprompt-related baseline. Finally, we individually examine each of the key prompt engineering–specific information reported across papers and find that many studies neglect to explicitly mention them, posing a challenge for advancing prompt engineering research.

**Conclusions:**

In addition to reporting on trends and the scientific landscape of prompt engineering, we provide reporting guidelines for future studies to help advance research in the medical field. We also disclose tables and figures summarizing medical prompt engineering papers available and hope that future contributions will leverage these existing works to better advance the field.

## Introduction

In recent years, the development of large language models (LLMs) such as GPT-3 has disrupted the field of natural language processing (NLP). LLMs have demonstrated capabilities in processing and generating human-like text, with applications ranging from text generation and translation to question answering and summarization [1]. However, harnessing the full potential of LLMs requires careful consideration of how input prompts are formulated and optimized [2].

Input prompts denote a set of instructions provided to the LLM to execute a task. Prompt engineering, a term coined to describe the strategic design and optimization of prompts for LLMs, has emerged as a crucial aspect of leveraging these models. By crafting prompts that effectively convey tasks or queries, researchers and practitioners can guide LLMs to improve the accuracy and pertinence of responses. The literature defines prompt engineering in various ways: it can be regarded as a prompt structuring process that enhances the efficiency of an LLM to achieve a specific objective [3] or as the mechanism through which LLMs are programmed by prompts [4]. Prompt engineering encompasses a plethora of techniques, often separated into distinct categories such as output customization and prompt improvement [4]. Existing prompt paradigms are presented in more detail in the Methods section.

In the realm of medical NLP, significant advancements have been made, such as the release of LLMs specialized in medical language and the availability of public medical data sets, including in languages other than English [5]. The unique intricacies of medical language, characterized by its terminological precision, context sensitivity, and domain-specific nuances, demand a dedicated focus and exploration of NLP in health care research. Despite these imperatives, to our knowledge, there is currently no systematic review analyzing prompt engineering applied to the medical domain.

The aim of this scoping review is to shed light on prompt engineering, as it is developed and used in the medical field, by systematically analyzing the literature in the field. Specifically, we examine the definitions, methodologies, techniques, and outcomes of prompt engineering across various NLP tasks. Methodological strengths, weaknesses, and limitations of the current wave of experimentation are discussed. Finally, we provide guidelines for comprehensive reporting of prompt engineering–related studies to improve clarity and facilitate further research in the field. We aspire to furnish insights that will inform both researchers and users about the pivotal role of prompt engineering in optimizing the efficacy of LLMs. By gaining a thorough understanding of the current landscape of prompt engineering research, we can pinpoint areas warranting further investigation and development, thereby propelling the field of medical NLP forward.

## Methods

### Study Design

Our scoping review was conducted following the PRISMA-ScR (Preferred Reporting Items for Systematic Reviews and Meta-Analyses extension for Scoping Reviews) guidelines for scoping reviews (available in Multimedia Appendix 1). In this review, we use terminology to denote emerging technical concepts that lack consensus definitions. We propose the following definitions based on previous use in the literature:

- LLM: Object that models language and can be used to generate text by receiving large-scale language modeling pretraining (Luccioni and Rogers [6] define an arbitrary threshold at 1 billion tokens of training data). An LLM can be adapted to downstream tasks through transfer learning approaches such as fine-tuning or prompt-based techniques. Following the study of Thirunavukarasu et al [7] of models for the medical field, we include Bidirectional Encoder Representations From Transformers (BERT)–based and GPT-based models in this definition, although Zhao et al [8] place BERT models in a separate category.
- Fine-tuning: Approach in which the weights of the pretrained LLM are retrained on new samples. The additional data can be labeled and designed to adapt the LLM to a new downstream task.
- Prompt design (PD) [1,2]: Manually building a prompt (named manual prompt or hard prompt), tailored to guide the LLM toward resolving the task by simply predicting the most probable continuity of the prompt. The prompt is usually a set of task-specific instructions, occasionally featuring a few demonstrations of the task.
- Prompt learning (PL) [3]: Manually building a prompt and passing it to an LLM, trained via the masked language modeling (MLM) objective, to predict masked tokens. The prompt often features masked tokens, over which the LLM makes predictions. Those are then projected as predictions for a new downstream task. This approach is also referred to as prompt-based learning.
- Prompt tuning (PT) [9]: Refers to the LLM prompting where part or all the prompt is a trainable vectorial representation (known as continuous prompt or soft prompt) that is optimized with respect to the annotated instances.

**Figure 1:**
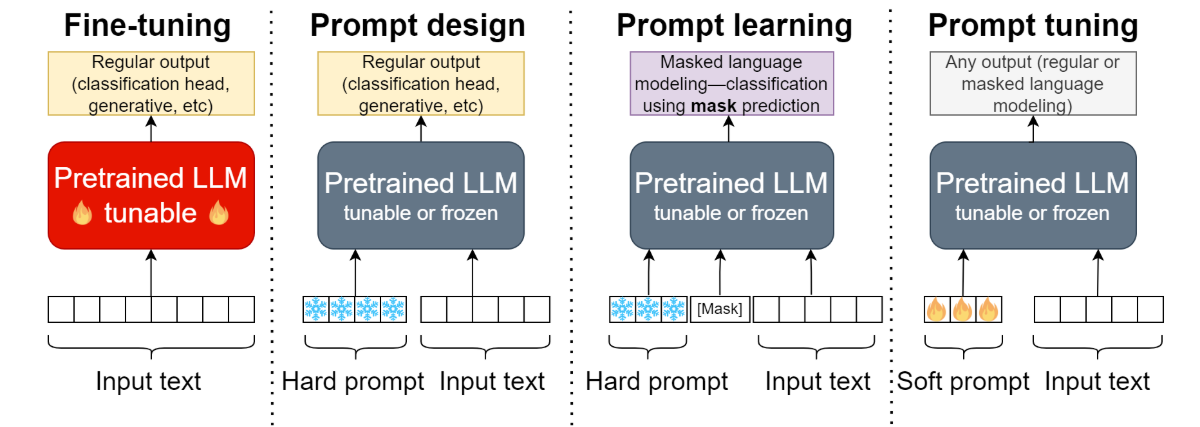
Illustration of traditional fine-tuning and the 3 prompt-based paradigms (the fire logo represents trainable parameters, and the flake logo illustrates frozen parameters). LLM: large language model.

Figure 1 illustrates the 4 approaches described above.

### Inclusion and Exclusion Criteria

Studies were included if they met the following criteria: focus on prompt engineering, involvement of at least 1 LLM, relevance to the medical field (biomedical or clinical), pertaining to text-based generation (excluding vision-related prompts), and not focusing on prompting for academic writing purposes. Furthermore, as most of the first studies about prompt engineering emerged in 2022 [2], we added the following constraint: the publication date should be later than 2021.

### Screening Process

The initial set of papers retrieved from the searches underwent screening based on titles, abstracts, and keywords. The search strategy is described in Multimedia Appendix 2. Screening was performed by 2 reviewers (JZ and MN), working in a double-blind process. Interannotator agreement was calculated, with conflicts resolved through discussion.

### Data Synthesis

We extracted information on prompt paradigms (PD, PL, and PT), involved LLMs, data sets used, studied language, domain (biomedical or clinical), medical subfield (if any), mentioned prompt engineering techniques, computational complexity, baselines, relative performances, and key findings. Additionally, we extracted journal information and noted instances of PD or PL or PT terminology misuse. Details are available in Multimedia Appendix 3. Finally, we compile a list of recommendations based on the positive or negative trends we identify from the selected papers.

## Results

### Screening Results

**Figure 2:**
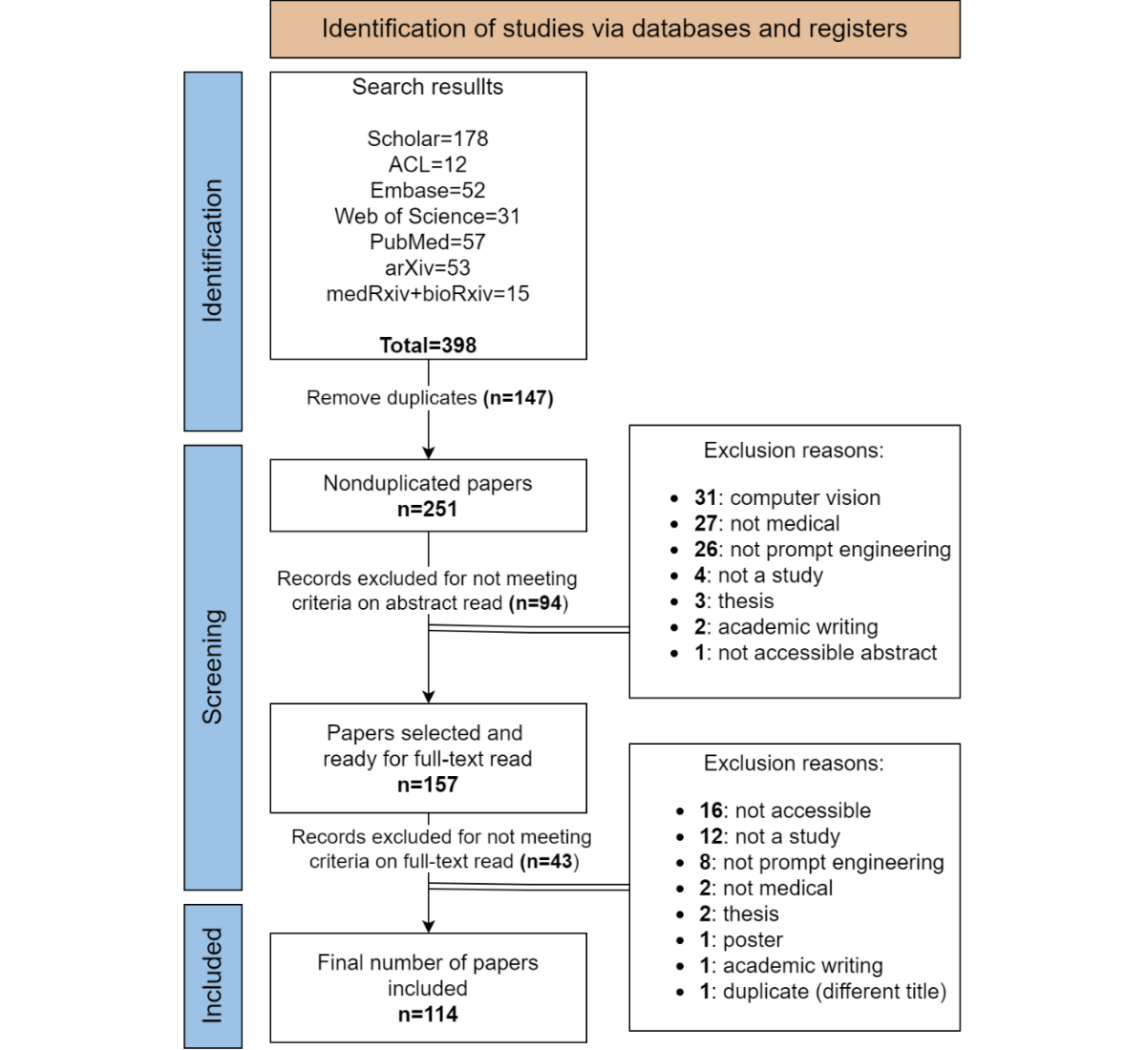
PRISMA (Preferred Reporting Items for Systematic Reviews and Meta-Analyses) flow diagram for the review process.

The systematic search across sources yielded 398 papers. Following the removal of duplicates, 251 papers underwent screening based on title, abstract, and keywords, leading to the exclusion of 94 studies. During this first screening step, 33 conflicts were identified and resolved among the annotators, resulting in an interannotator agreement of 86.8% (n=218). Subsequently, 157 studies remained, and full-text copies were retrieved and thoroughly screened. This process culminated in the inclusion of a total of 114 papers in this scoping review. The detailed process of study selection is shown in Figure 2. Among the selected papers, 13 are from clinical venues, 33 are from medical informatics sources, 31 are from computer science publications, and 4 are from other sources. Notably, 33 of them are preprints.

### Prompt Paradigms and Medical Subfields

Table 1 depicts the number of papers identified within each prompt paradigm along with their associated medical subfields. Some papers may simultaneously involve several (up to 2 in this review) prompt paradigms. Notably, PD emerged as the predominant category, with a total of 78 papers. These papers spanned across various medical fields, with a greater emphasis on clinical (including specialties) rather than biomedical disciplines. The screening yields 29 PL papers and 19 PT papers, with both paradigms maintaining a balanced distribution between biomedical and clinical domains. However, it is noteworthy that unlike PL and PT, PD encompassed a much broader spectrum of clinical specialties, with a particular interest in psychiatry.

**Table 1:** Paper distribution by prompt category and medical subfield, with corresponding references.

Prompt paradigm and domain of the topic		References
**Prompt design (78)**		
	Biomedical (17)	[10-26]
	Medical licensing examination (12)	[27-38]
	Clinical (general) (15)	[39-53]
	Psychiatry (10)	[28,54-62]
	Oncology (5)	[63-67]
	Cardiology (4)	[68-71]
	Ophthalmology (3)	[72-74]
	Neurology (3)	[69,75,76]
	Orthopedics (2)	[77,78]
	Clinical trials (2)	[79,80]
	Intensive care (2)	[69,81]
	Geriatrics (2)	[75,76]
	Radiology (2)	[31,82]
	Nuclear medicine (1)	[29]
	Hepatology (1)	[83]
	Endocrinology (1)	[84]
	Plastic surgery (1)	[85]
	Gastroenterology (1)	[32]
	Genetics (1)	[86]
	Nursing (1)	[87]
**Prompt learning (29)**		
	Biomedical (13)	[88-100]
	Clinical (general) (15)	[41,47,101-113]
	Psychiatry (1)	[114]
**Prompt tuning (19)**		
	Biomedical (9)	[16,20,26,90,91,95,98,115,116]
	Clinical (general) (6)	[101,105,110,117-119]
	Oncology (2)	[120,121]
	Psychiatry (1)	[122]
	Medical insurance (1)	[123]

### Terminology Use

In our review, the consistency of terminology use around prompt engineering was investigated, particularly concerning its 3 paradigms: PD, PL, and PT. Across the papers, we meticulously tracked instances where the terminology was applied differently to the definitions used in the literature and described in the introduction. Notably, PL was used to refer to PD 4 times [12,13,67,86] and PT once [119], while PT was used 5 times to describe PL [88,96,97,99,114] and twice for PD [23,43]. Terminology inconsistencies were identified in only 12 studies. Consequently, while there remains some degree of inconsistency, a significant majority of 102 papers adhered to the definitions identified as commonly used terminology.

### Language of Study

Considering the latest developments in NLP research encompassing languages beyond English [124], reporting the language of study is crucial. Several papers do not explicitly state the language of study. In some cases, the language can be inferred from prompt illustrations or examples. In the least informative cases, only the data set of the study is disclosed, indirectly hinting at the language.

Table 2 illustrates the language distribution among the selected papers, noting whether languages are explicitly mentioned, implicitly inferred from prompt illustrations, or simply not stated but implied from the used data set. The language used in 2 papers [60,68] remains unknown.

**Table 2:** Frequency distribution of papers across various languages. The table also depicts the frequency distribution across venues for papers studying English (N=114).

Language and type of venue		Stated^a^, n (%)	Inferred^b^, n (%)	Not stated^c^, n (%)	Total, n (%)
**English**					
	All	37 (32.5)	48 (42.1)	11 (9.6)	96 (84.2)
	Medical informatics	16 (14)	9 (7.9)	2 (1.8)	27 (23.7)
	Computer science	8 (7)	18 (15.8)	1 (0.9)	27 (23.7)
	Preprint	9 (7.9)	12 (10.5)	5 (4.4)	26 (22.8)
	Clinical	1 (0.9)	8 (7)	3 (2.6)	12 (10.5)
	Other	3 (2.6)	1 (0.9)	0 (0)	4 (3.5)
**Chinese**					
	All	18 (15.8)	0 (0)	0 (0)	18 (15.8)
**French**					
	All	3 (2.6)	0 (0)	0 (0)	3 (2.6)
**Dutch**					
	All	3 (2.6)	0 (0)	0 (0)	3 (2.6)
**Japanese**					
	All	2 (1.8)	0 (0)	0 (0)	2 (1.8)
**Portuguese**					
	All	2 (1.8)	0 (0)	0 (0)	2 (1.8)
**Italian**					
	All	2 (1.8)	0 (0)	0 (0)	2 (1.8)
**Spanish**					
	All	2 (1.8)	0 (0)	0 (0)	2 (1.8)
**Korean**					
	All	0 (0)	0 (0)	1 (0.9)	1 (0.9)
**Basque**					
	All	1 (0.9)	0 (0)	0 (0)	1 (0.9)
**German**					
	All	1 (0.9)	0 (0)	0 (0)	1 (0.9)
**Swedish**					
	All	1 (0.9)	0 (0)	0 (0)	1 (0.9)
**Polish**					
	All	1 (0.9)	0 (0)	0 (0)	1 (0.9)
**Vietnamese**					
	All	1 (0.9)	0 (0)	0 (0)	1 (0.9)
**Unknown**					
	All	0 (0)	0 (0)	2 (1.8)	2 (1.8)

^a^Stated in the paper.

^b^Inferred from prompt figures and examples.

^c^Inferred from the data set.

Notably, English dominates with 84.2% (n=96) of the selected papers, followed by Chinese at 15.7% (n=18). Then, the other languages are relatively rare, often appearing in studies featuring multiple languages. It is worth mentioning that languages besides English are usually explicitly stated, with the exception of a paper studying Korean [63]. In total, the language had to be inferred from prompt figures and examples in 48 papers, all in English.

### Choice of LLMs

Given the diverse array of LLMs available, spanning general or medical, open-source or proprietary, and monolingual or multilingual models, alongside various architectural configurations (encoder, decoder, or both), our study investigates LLM selection across prompt paradigms.

**Figure 3:**
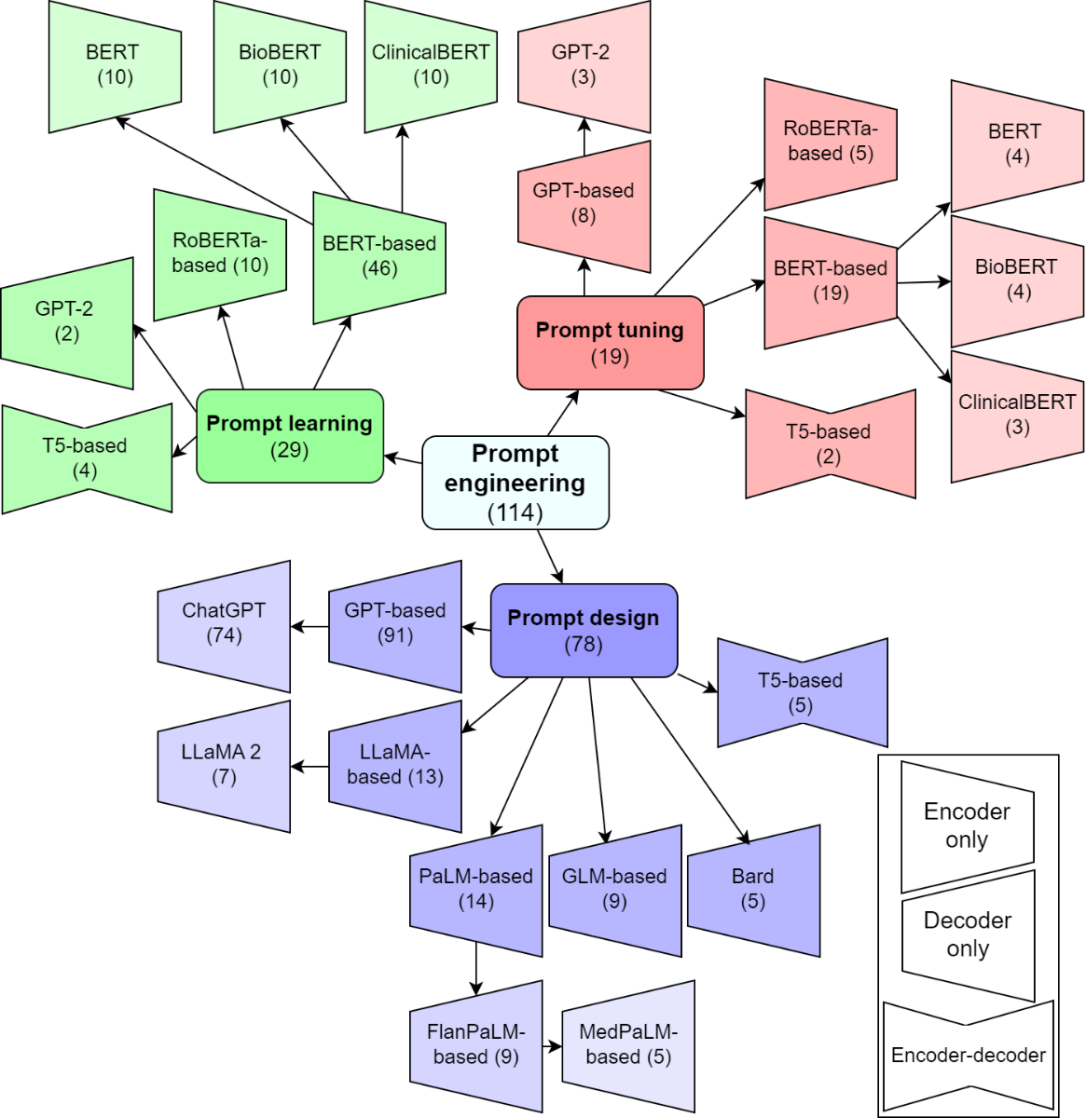
Involved large language models in the prompt engineering studies, covering all prompt paradigms. The number of studies that fit in a node is shown in parentheses. BERT: Bidirectional Encoder Representations From Transformers; RoBERTa: Robustly Optimized BERT Pre-training Approach; T5: Text-to-Text Transfer Transformer.

Figure 3 outlines prevalent LLMs categorized by prompt paradigms, though it is not exhaustive and only includes commonly encountered architectures. For example, while encoder-decoder models are absent in PT in Figure 3, there are a few instances where they are used [95,110].

ChatGPT’s popularity in PD is unsurprising, given its accessibility. Models from Google, PaLM, and Bard (subsequently rebranded Gemini), all falling under closed models, are also prominent. Among open-source instruct-based LLMs, fewer are used, notably those based on LLaMA-2 with 7 occurrences.

In PL, encoder models, those following the BERT architecture, dominate, covering both general and specialized variants. There are occasional uses of decoder models like GPT-2 in PL-based tasks [103,105]. PT involves all model types, with a preference toward encoders. Further details on the models used are available in Multimedia Appendix 3.

### Topic Domain and NLP Task Trends

**Figure 4:**
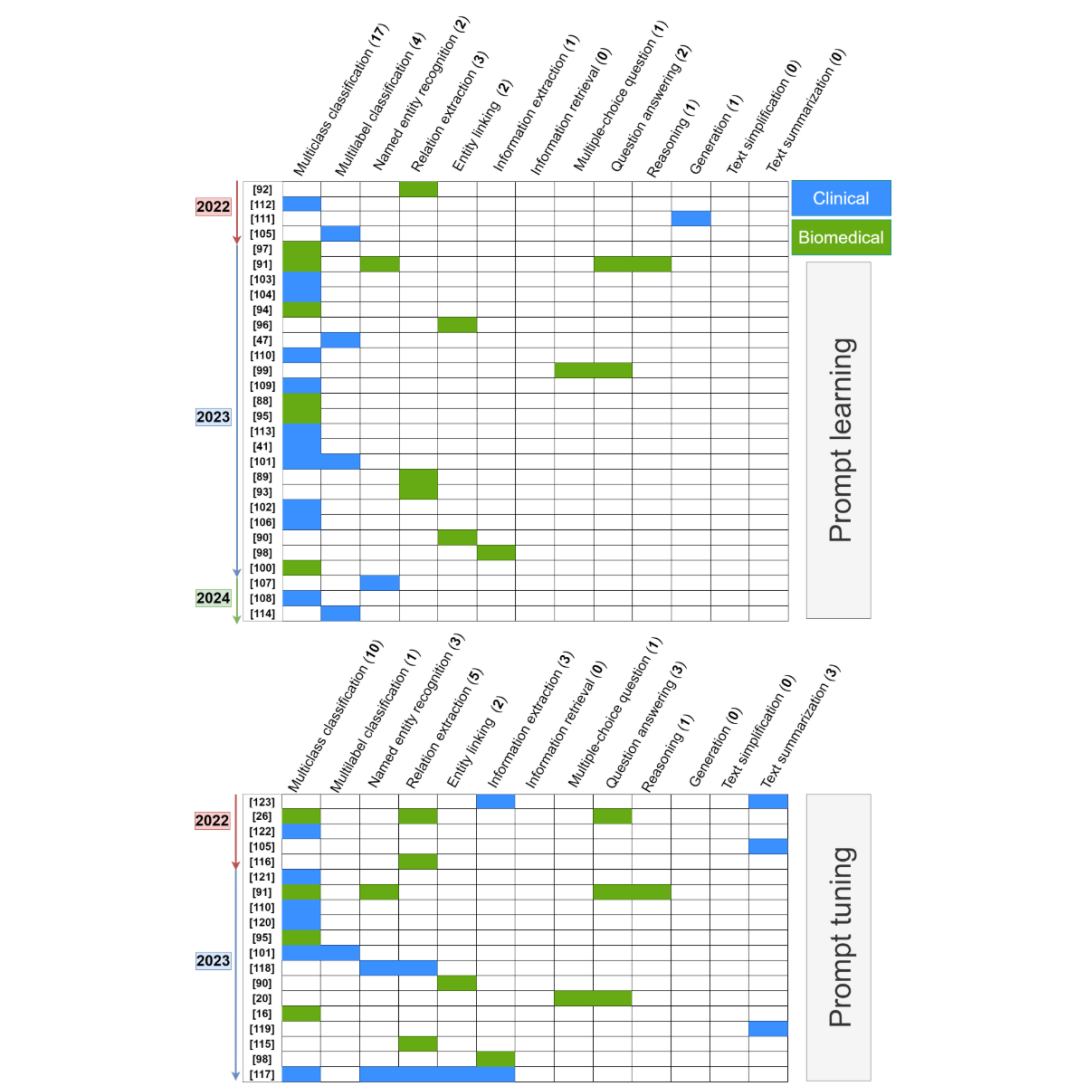
Overview of selected prompt learning and prompt tuning papers, showcasing natural language processing tasks alongside their topic domain (it includes tasks, such as text simplification, where none of the selected papers specifically focused on these tasks). Numbers within square brackets are reference citations.

Figure 4 [16,20,26,41,47,88-123] illustrates the target tasks used in the PL and PT papers. PL-focused papers predominantly address classification-based tasks such as text classification, named entity recognition, and relation extraction, with text classification being particularly prominent. This aligns with the nature of PL, which centers around an MLM objective. Among other tasks, a study based on text generation [111] makes use of PL to predict masked tokens from partial patient records, aiming to generate synthetic electronic health records. Conversely, PT papers tend to exhibit a slightly broader range of tasks.

**Figure 5:**
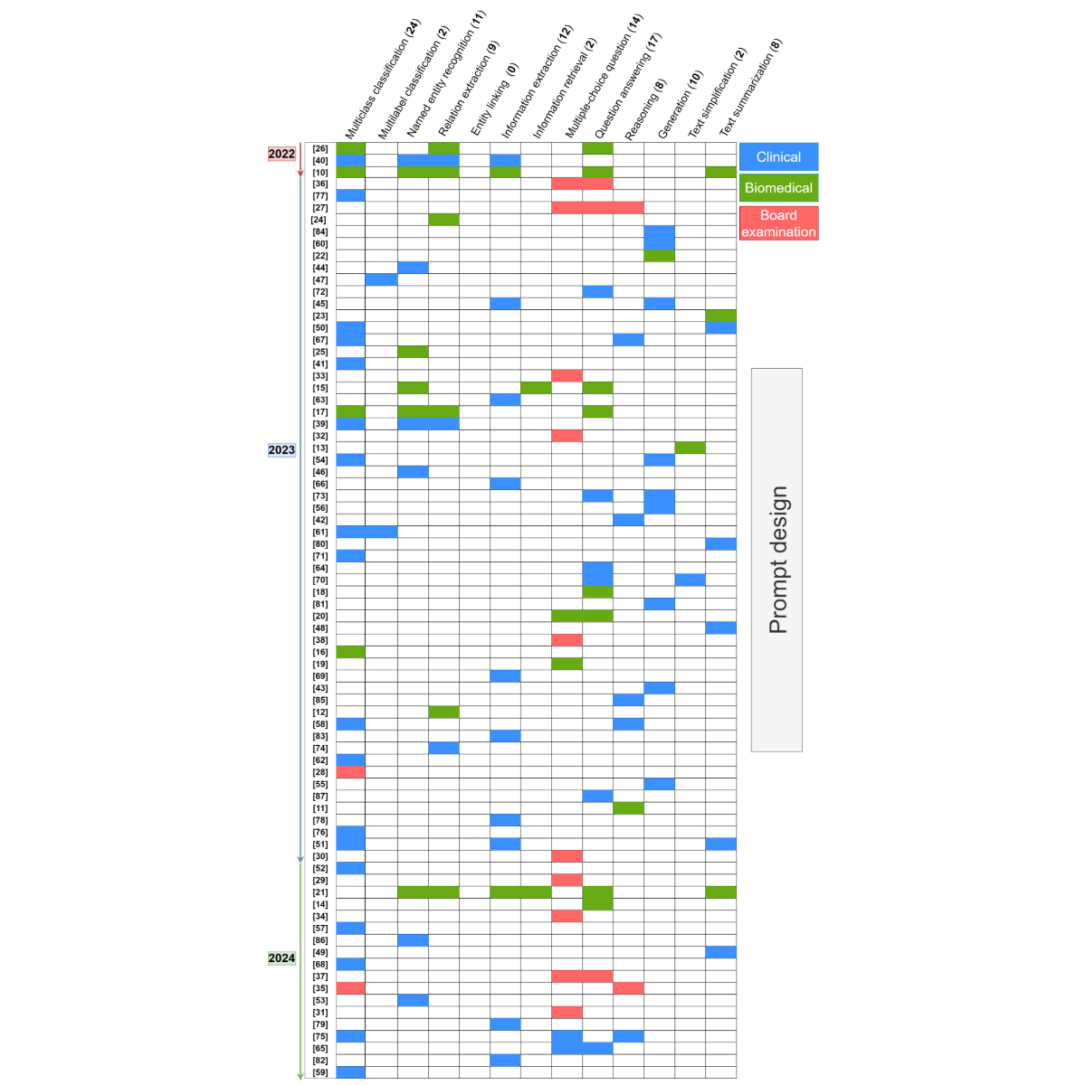
Overview of selected prompt design papers, showcasing natural language processing tasks alongside their topic domain. Numbers within square brackets are reference citations.

Figure 5 [10-87] presents the same analysis for PD-based papers. Unlike PL and PT, a prominent trend observed is that several studies focus on real-world board examinations. Notably, these studies predominantly center around tasks involving answering multiple-choice questions (MCQs). It is worth noting that although MCQs might be cast as a classification task, in practice, it is cast as a generation task using causal LLMs. It is interesting to note that none of the selected PD papers propose the task of entity linking, despite the clear opportunity of leveraging LLMs’ in-context learning ability for medical entity linking.

### Prompt Engineering Techniques

We extensively investigated the used prompt techniques: among PD papers, 49 studies used zero-shot prompting, 23 used few-shot prompting, and 10 used one-shot prompting. Few shot tends to outperform in MCQs, but its advantage over zero shot is inconsistent in other NLP tasks. We propose a comprehensive summary of the existing techniques in Table 3.

As shown in Table 3, chain-of-thought (CoT) prompting [2] stands as the most common technique, followed by the persona pattern. In medical MCQs, various attempts with CoT can lead to different reasoning pathways and answers. Hence, to improve accuracy, 2 studies [19,20] used self-consistency, a method involving using multiple CoT prompts and selecting the most frequently occurring answer through voting.

Flipped interaction was used for simulation tasks, such as doctor-patient engagement [60] or to provide clinical training to medical students [81]. Emotion enhancement was applied in mental health contexts [58,60], allowing the LLM to produce emotional statements.

More innovative prompt engineering techniques include k-nearest neighbor few-shot prompting [19] and pseudoclassification prompting [78]. The former uses the k-nearest neighbor algorithm to select the k-closest examples in a large annotated data set based on the input before using them in the prompt, and the latter presents to the LLMs all possible labels, asking the model to respond with a binary output for each provided label. Despite its potential, tree-of-thoughts pattern use was limited, with only 1 instance found among the papers [77].

**Table 3:** Most recurrent prompt techniques found, with the corresponding description, template, and references.

Prompt techniques	Description	Prompt template examples	Count papers	References
Chain-of-thought (CoT)	Asking the large language model (LLM) to provide the reasoning before answering.	Basic CoT: “<Prompt>. Think step by step.” Another example of CoT: “Solve this math problem. E.g.: You have 3 apples and buy 2 more, how many apples do you have? Solution: Start with 3 apples. Buy 2 more apples. Total apples is 3 + 2 = 5. New problem: You have 5 oranges and give away 2, how many oranges do you have left?”	17	[11,19,20,27,29,32,33,35,39, 51,58,67,75,77,82,83,85]
Persona (role-defining)	Assigning the LLM a particular role to accomplish a task related to that role.	“Act as X (e.g. Act as a Physician, Act as a Psychiatrist, etc).”	10	[32,49,55,56,59-61,82,84,85]
Ensemble prompting	Using multiple independent prompts to answer the same question. The final output is decided by majority vote.	“Prompt1, Output1, Prompt2, Output2, […], Promptk, Outputk” Final output: Vote	4	[19,20,39,52]
Scene-defining	Simulating a scene related to the addressed task.	“you are in a hospital, in front of a patient ...”	3	[18,49,61]
Prompt-chaining	Separating a task into multiple subtasks, each resolved with a prompt.	“Prompt1->Output1, Output1+Prompt2 ->Output2, [...] Outputk-1+Promptk-> Outputk”	3	[37,80,84]
Flipped interaction	Making the LLM take the lead (eg, asking questions) and the user interacting with it passively.	“I would like you to ask me questions to achieve X. You should ask questions until <condition/goal> is met.”	2	[60,81]
Emotion enhancement	Making the LLM more or less expressing human-like emotions.	“You can have emotional fluctuations during the conversation.”	2	[58,60]
Prompt refinement	Using the LLM to refine the prompt such as translating the prompt or rephrasing it.	“Please translate in English / rephrase this prompt: <P>.”	2	[37,48]
Retrieval-augmented generation	Combining an information retrieval component with a generative LLM. Snippets extracted from documents are fed into the system along with the input prompt to generate an enriched output.	“<List of relevant Snippets> <Input Prompt>”	2	[18,54]
Self-consistency (CoT ensembling)	Ensemble prompting each prompt using CoT. Ideal if a problem has many possible reasoning paths.	“CoT_Pr1, Output1, CoT_Pr2, Output2, ..., CoT_Prk, Outputk” Final output: Vote	2	[19,20]

### Emerging Trends

**Figure 6:**
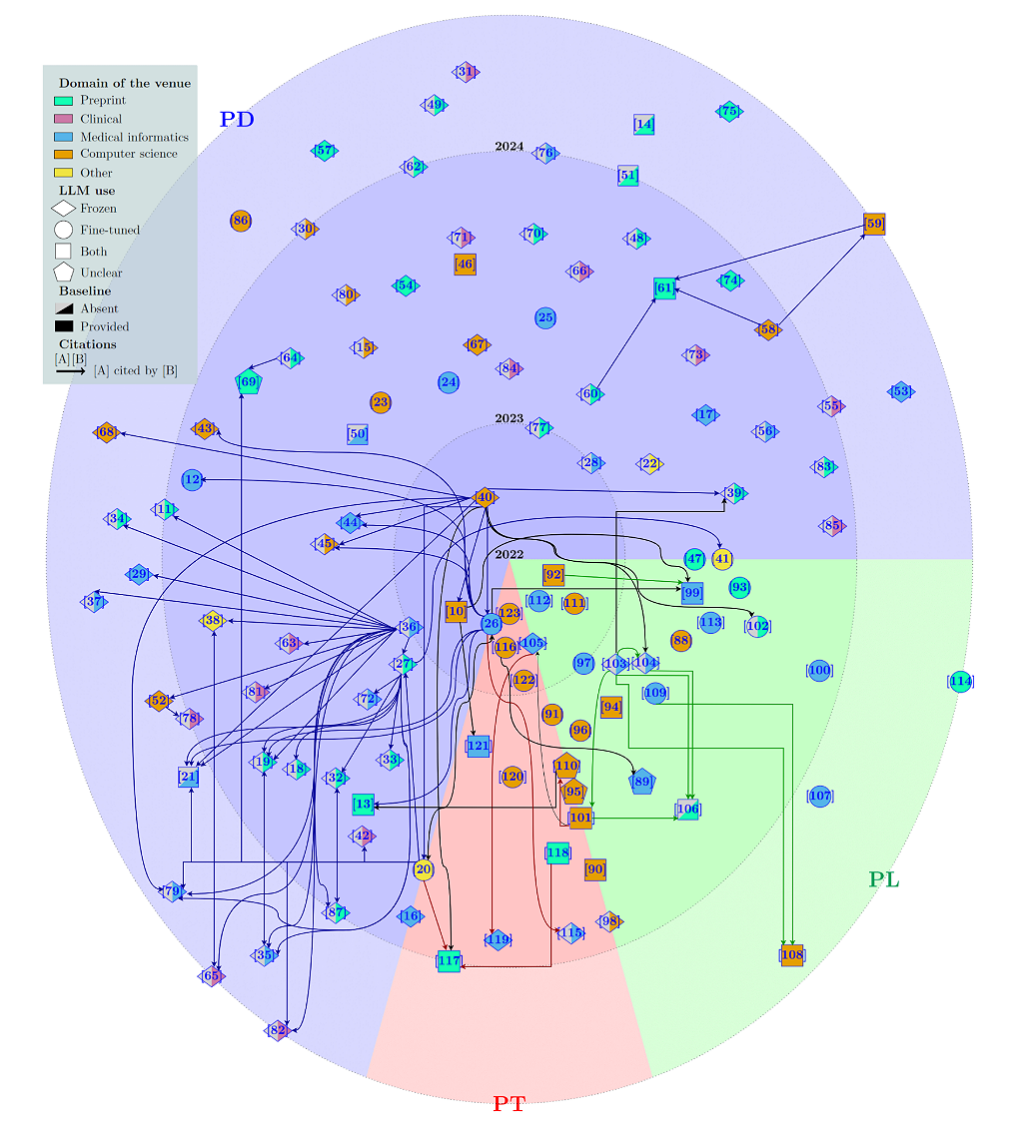
A chronological chart showing the selected papers across the 3 prompt-based paradigms. Papers are classified by different colors according to the venues in which they were published. Different shapes illustrate whether the LLM is fine-tuned, frozen, or both. Solid or striped color indicates whether authors used a nonprompt baseline (including humans) for evaluation. Arrows connecting 2 papers denote direct citations. The nodes in the border of PD, PL, or PT are studies proposing the 2 involved prompt engineering paradigms. Numbers within square brackets are reference citations. LLM: large language model; PD: prompt design; PL: prompt learning; PT: prompt tuning.

Figure 6 illustrates a chronological polar pie chart of selected papers and their citation connections, identifying five highly cited papers: (1) Agrawal et al [40] demonstrate GPT-3’s clinical task performance, especially in named entity recognition and relation extraction through thorough PD. (2) Kung et al [36] evaluate ChatGPT’s (GPT-3.5) ability for the United States Medical Licensing Examination, shortly after the public release of ChatGPT. (3) Singhal et al [20] introduce MultiMedQA and HealthSearchQA benchmarks. The paper also presents instruction PT for domain alignment, a novel paradigm that entails learning a soft prompt prior to the LLM general instruction, which is usually written as a hard prompt. Using this approach on FlanPaLM led to the development of Med-PaLM, improving question answering over FlanPaLM. (4) Nori et al [27] evaluate GPT-4 on the United States Medical Licensing Examination and MultiMedQA, surpassing previous state-of-the-art results, including GPT-3.5 and Med-PaLM. (5) Luo et al [26] release BioGPT, a fine-tuned variant of GPT-2 for biomedical tasks, achieving state-of-the-art results on 6 biomedical NLP tasks with suffix-based PT.

### Trends in PD

As shown in Figure 6, the PD paradigm presents multiple trends: all papers disseminated in clinical-based venues, and 27 of 33 (82%) of the encountered preprints adhere to this paradigm. Furthermore, we observed a significant focus on work involving frozen LLMs within the PD domain. This trend is likely due to the frequent use of ChatGPT in 74 instances, as depicted in Figure 3, despite OpenAI offering fine-tuning capabilities for the model. It is worth mentioning that 46 of 78 (59%) PD papers do not include any baseline, including human comparison. This gap will be further explored in a subsequent section.

### Trends in PL and PT

Among PL and PT papers, computer science and medical informatics are the most prevalent venues. Although PL has drawn attention to the idea of adapting the MLM objective to downstream tasks without needing to further update the LLM weights, many studies still opt to fine-tune their LLMs, with a nonnegligible amount of them evaluating in few-shot settings [89,92,93,112]. Unlike PD, PL and PT usually include a baseline, with it often being a traditional fine-tuning version of the evaluated model [92,93,95] to compare it against novel prompt-based paradigms. These studies came to a common conclusion, being that PL is a promising alternative to traditional fine-tuning in few-shot scenarios.

There are 2 ways for conducting PL: one involves filling in the blanks within a text, known as cloze prompts, while the other consists in predicting masked tokens at the end of the sequence, referred to as prefix prompts. A distinct advantage of the latter approach is its compatibility with autoregressive models, as they exclusively predict the appended masks. Among the 29 PL papers, 21 (72%) of them propose cloze prompts, while 15 (52%) use prefix prompting. The involved NLP tasks are well-distributed across these 2 prompt patterns. Another crucial component of PL is the verbalizer. As PL revolves around predicting masked tokens, classification-based tasks require mapping manually selected relevant tokens to each class (manual verbalizer). Alternatively, some studies propose a soft verbalizer, akin to soft prompts, which automatically determines the most relevant token embedding for each label through training. Of the 29 PL papers selected, 16 (55%) studies explicitly mention the use of a manual verbalizer, while 2 explored both verbalizers to assess performance [101,110]. Only 1 exclusively used a soft verbalizer [89]. Another study does not use any verbalizer, as it focuses on generating synthetic data by filling the blanks [111]. Notably, 8 (28%) studies did not report any mention regarding the verbalizer methodology.

Hard prompts, which are related to PD and PL, involve manually crafted prompts. Regarding PT, optimal prompts are attainable through soft prompting (ie, prompts that are trained on a training data set), yet, determining the appropriate soft prompt length remains obscure. In total, 5 of 19 (26%) PT studies tried various soft prompt lengths and reported their corresponding performances [26,105,118,119,122]. While there is no definitive optimal prompt length, a trend emerges: optimal soft prompt length typically exceeds 10 tokens. Surprisingly, 8 (42%) papers omit reporting the soft prompt length. Regarding the placement of soft prompts in relation to the input and the mask, consensus is lacking. A total of 5 (26%) papers prepend the soft prompt at the input’s outset, while 4 (21%) append it as a suffix. One paper uses both strategies in a single prompt template [95]. Some innovative methods involve inserting a single soft prompt for each entity that needs to be identified in entity-linking tasks or using token-wise soft prompts, where each token in the textual input is accompanied by a distinct soft prompt. The position of soft prompts remains unreported in 5 (26%) studies. Finally, according to the 6 (32%) studies that used mixed prompts [90,91,95,101,105,110] (a combination of hard and soft prompts), it has consistently been reported that mixed prompts lead to a better performance than hard prompts alone.

### Baseline Comparison

Only 62 of the screened papers reported comparisons to established baselines. These include traditional deep learning approaches (eg, fine-tuning approach), classical machine learning algorithms (eg, logistic regression), naive systems (eg, majority class), or human annotation. The remaining papers solely explored prompt-related solutions, without including baseline comparisons. Tables 4-6 traces the presence of a nonprompt baseline among different prompt categories (Table 4), papers sources (Table 5), and NLP tasks addressed (Table 6).

**Table 4:** Baseline reports among prompt categories (N=114)^a^.

Prompt category	No baseline, n (%)	Higher, n (%)	Similar, n (%)	Lower, n (%)	Total, n (%)
Prompt design	48 (42.1)	13 (11.4)	4 (3.5)	13 (11.4)	78 (68.4)
Prompt learning	5 (4.4)	19 (16.7)	3 (2.6)	2 (1.8)	29 (25.4)
Prompt tuning	3 (2.6)	11 (9.6)	2 (1.8)	3 (2.6)	19 (16.7)

^a^Higher or lower indicates that the performance of the proposed prompt-based approach is higher or lower than the baseline.

**Table 5:** Baseline reports among venues (N=114)^a^.

Type of venue	No baseline, n (%)	Higher, n (%)	Similar, n (%)	Lower, n (%)	Total, n (%)
Medical informatics	13 (11.4)	16 (14)	2 (1.8)	2 (1.8)	33 (28.9)
Computer science	7 (6.1)	12 (10.5)	3 (2.6)	9 (7.9)	31 (27.2)
Preprint	21 (18.4)	6 (5.3)	1 (0.9)	5 (4.4)	33 (28.9)
Clinical	13 (11.4)	0 (0)	0 (0)	0 (0)	13 (11.4)
Other	1 (0.9)	2 (1.8)	0 (0)	1 (0.9)	4 (3.5)

^a^Higher or lower indicates that the performance of the proposed prompt-based approach is higher or lower than the baseline.

**Table 6:** Baseline reports among addressed NLP^a^ tasks (N=114)^b^.

NLP task	No baseline, n (%)	Higher, n (%)	Similar, n (%)	Lower, n (%)	Total, n (%)
Text classification	13 (11.4)	18 (15.8)	4 (3.5)	11 (9.6)	46 (40.4)
Question answering	13 (11.4)	3 (2.6)	1 (0.9)	2 (1.8)	19 (16.7)
Relation extraction	3 (2.6)	10 (8.8)	0 (0)	3 (2.6)	16 (14)
Information extraction	10 (8.8)	3 (2.6)	0 (0)	2 (1.8)	15 (13.2)
Multiple-choice question	10 (8.8)	3 (2.6)	1 (0.9)	1 (0.9)	15 (13.2)
Named entity recognition	4 (3.5)	5 (4.4)	1 (0.9)	5 (4.4)	15 (13.2)
Text summarization	7 (6.1)	3 (2.6)	0 (0)	1 (0.9)	11 (9.6)
Reasoning	5 (4.4)	3 (2.6)	0 (0)	1 (0.9)	9 (7.9)
Generation	5 (4.4)	2 (1.8)	0 (0)	1 (0.9)	8 (7)
Entity linking	0 (0)	3 (2.6)	0 (0)	0 (0)	3 (2.6)
Coreference resolution	1 (0.9)	1 (0.9)	0 (0)	1 (0.9)	3 (2.6)
Decision support	2 (1.8)	0 (0)	0 (0)	1 (0.9)	3 (2.6)
Conversational	3 (2.6)	0 (0)	0 (0)	0 (0)	3 (2.6)
Text simplification	1 (0.9)	0 (0)	0 (0)	1 (0.9)	2 (1.8)

^a^NLP: natural language processing.

^b^Higher or lower indicates that the performance of the proposed prompt-based approach is higher or lower than the baseline.

Nonprompt-related baselines are often featured in studies focused on PL and PT but not PD. Additionally, PL and PT have a tendency to perform better than their respective reported baselines, PD tends to report less conclusive results. More specifically, among the 22 papers using either PL or PT with an identical fine-tuned model as a baseline, 17 indicate superior performance with the prompt-based approach, 3 observed comparable performance, and 2 studies noted inferior performance.

Significantly, papers from computer science venues tend to include more state-of-the-art baselines than those from medical informatics and clinical venues. Specifically, all 13 papers reviewed from clinical venues did not use any nonprompt baselines. Furthermore, there appears to be no consistent link between the type of NLP tasks and the omission of baselines, indicating that the decision to include baselines is more influenced by the evaluation methodology than by feasibility.

### Prompt Optimization

Numerous studies in the literature highlight the few-shot learning capabilities of LLMs, often referred to as “few-shot prompting,” wherein they demonstrate proficiency in executing tasks with minimal demonstrations provided, typically through text prompts. However, it is crucial to acknowledge that the annotation cost associated with such frameworks might extend beyond the few annotated demonstrations within the prompt. Many studies claiming to explore few-shot or zero-shot learning through prompt engineering rely on extensive annotated validation data sets to refine PD and formulation. This is, for example, the case in the paper that popularized the term “few-shot learning” [1]. Among the 45 analyzed papers concentrating on few-shot or zero-shot learning, 5 explicitly detail the optimization of prompt formulation using extensive validation data sets. Conversely, 18 of these papers either do not engage in prompt optimization or test various prompts and document all results. Notably, 22 papers present results using only 1 prompt choice, without clarifying whether this choice was made thanks to additional validation data sets.

## Discussion

### Summary of the Findings

This scoping review aimed to map the current landscape of medical prompt engineering, identifying key themes, gaps, and trends within the existing literature. The primary findings of this study reveal a greater prevalence of PD over PL and PT, with ChatGPT dominating the PD domain. Additionally, many studies omit nonprompt-based baselines, do not specify the language of study, or exhibit a lack of consensus in PL (prefix vs cloze prompt) and PT settings (soft prompt lengths and positions). English is notably dominant as the language of study. These findings suggest that while the field is emerging, there is a pressing need for improved research practices.

### Costs, Infrastructure, and LLMs in Clinical Settings

Prompt engineering techniques enable competitive performance in scenarios with limited or no resources as well as in environments with low-cost computing infrastructure. As hospital data and infrastructure are often found in this scenario, these approaches hold great promise in the clinical field. Figure 6 shows the absence of PL- and PT-related works in clinical journals. This trend may stem from the widespread accessibility of ChatGPT, favoring PD-focused investigations. Despite efforts like OpenPrompt [125] to facilitate PL and PT works, the programming barrier likely deters clinical practitioners. Surprisingly, 7 papers use ChatGPT with sensitive clinical data. Despite the recent availability of ChatGPT Enterprise in GPT-4 for secure data handling, it is apparent that most of these studies have not used this feature since they used GPT-3.5. Limited use of local LLMs, especially LLaMA-based, suggests a need for their increased adoption in future clinical PD studies. The lack of local LLMs may be due to clinicians’ limited computational infrastructure.

### Prompt Engineering Techniques Effectiveness in Medical Research

In documented prompt engineering techniques, the effectiveness of few-shot prompting compared to zero shot varies by task and scenario. However, CoT shows superior reasoning performance, compelling LLMs to present reasoning pathways and consistently outperforming zero-shot and few-shot methods across PD studies. Its ensemble-based variant, self-consistency, consistently outperforms CoT. Despite the persona pattern’s frequent use, there is a lack of ablation studies on its impact on medical task performance, with only 1 paper reporting negligible improvement [61]. Prompt engineering is an emerging field of study that still needs to prove its efficacy. However, almost half of the papers focused only on prompt engineering and failed to report any nonprompt-related baseline performance, despite the availability of such baselines for the addressed NLP tasks. On the whole, the results are far from being systematically in favor of LLM-based methods, greatly attenuating the impression of a technological breakthrough that is generally commented on. Selecting a baseline remains a necessary step toward understanding the actual impact of prompt engineering.

### Bender Rule

Regarding the languages, while Table 2 shows the dominance of English in medical literature, many papers studying English fail to explicitly mention the language of study. This oversight is more prevalent in computer science and clinical venues, whereas medical informatics exhibits a more favorable trend, as validated by a chi-square test yielding a *P* value of .02 (Table S1 in Multimedia Appendix 2). Notably, languages such as Chinese are consistently mentioned across the 18 selected papers. However, the Bender rule, namely “always name the language(s) you are working on,” seems to be well respected for languages other than English. This finding has already been documented for NLP research in general [126].

### Fine-Tuning Versus Prompt-Based Approaches

While traditional LLM fine-tuning remains a viable method for various NLP tasks, PL and PT are competitive alternatives to fine-tuning, particularly in resource-constrained and low computational scenarios. PL, leveraging predefined prompts to guide model behavior, offers an efficient approach in low-to-no resource environments. Conversely, PT emerges as a viable solution in low computational scenarios, as it requires substantially fewer trainable parameters compared to traditional fine-tuning approaches. Since both prompt-based approaches do not require the LLM to be further trained, they are less prone to catastrophic forgetting [127].

### Recommendations for Future Medical Prompt–Based Studies

For future research in prompt engineering, we propose several recommendations aimed at improving research quality, reporting, and reproducibility. From this review, we identified several trends such as the computational advantages or the lack of evaluations on baselines with a lack of ablation studies to evaluate the performance of the prompting strategies. Some studies do not clearly mention the prompt engineering choices they made. For instance, in PL, choices range from using cloze to prefix prompting and from using manual to soft verbalizer. Similarly, PT is characterized by configurations of soft prompts, such as the length and the positions. To clarify these distinctions and enhance methodological transparency and reproducibility in future research, we have developed reporting guidelines available in Textbox 1. Adhering to these reporting guidelines will contribute to advancing prompt engineering methodologies and their practical applications in the medical field.

**Detailed reporting guidelines for future prompt engineering studies.**

**General reporting recommendations**

- For sensitive data, local large language models (LLMs) should be preferred to the ones that use an application programming interface or a web service.
- The language of the study used should be explicitly stated.
- The mention of whether the LLM undergoes fine-tuning should be made explicit.
- The prompt optimization process and results should be documented to ensure transparency, whether it is through different tested manual prompts or through a validation data set.
- The terms “few-shot,” “one-shot,” and “zero-shot” should not be used in settings where the prompts have been optimized on annotated examples.
- Experiments should include baseline comparisons or at least mention existing results, particularly when data sets originate from previous medical challenges or benchmarks.

**Specific to prompt learning and prompt tuning**

- Concepts (such as prompt learning and prompt tuning) should be defined and used consistently with the consensus.
- In prompt learning experiments, the verbalizer used (soft and hard) should be explicitly specified, or a clear justification should be provided if the verbalizer is omitted. Additionally, whether the prompt template follows the cloze or the prefix format should be mentioned.
- In prompt tuning experiments, authors should provide details on soft prompt positions, length, and any variations tested, such as incorporating hard or mixed prompts, as part of the ablation study.

### Limitations

A limitation was the large number of papers retrieved during the initial search, which was addressed by limiting the search scope to titles, abstracts, and keywords. Furthermore, since some studies may perform prompt engineering techniques without mentioning any of the 4 prompt-related expressions used in the queries, they might be missed by our searches.

### Conclusions

Medical prompt engineering is an emerging field with significant potential for enhancing clinical applications, particularly in resource-constrained environments. Despite the promising capabilities demonstrated, there is a pressing need for standardized research practices and comprehensive reporting to ensure methodological transparency and reproducibility. Consistent evaluation against nonprompt-based baselines, prompt optimization documentation, and prompt settings reporting will be crucial for advancing the field. We hope that a better adherence to the recommended guidelines, in Textbox 1, will improve our understanding of prompt engineering and enhance the capabilities of LLMs in health care.

## Appendix

Multimedia Appendix 1 PRISMA-ScR (Preferred Reporting Items for Systematic Reviews and Meta-Analyses extension for Scoping Reviews) checklist.

Multimedia Appendix 2 Search strategy and statistical analysis.

Multimedia Appendix 3 Reading notes and details of the reviewed papers.

## Abbreviations

BERT: Bidirectional Encoder Representations From Transformers
CoT: chain-of-thought
LLM: large language model
MCQ: multiple-choice question
MLM: masked language modeling
NLP: natural language processing
PD: prompt design
PL: prompt learning
PRISMA-ScR: Preferred Reporting Items for Systematic Reviews and Meta-Analyses extension for Scoping Reviews
PT: prompt tuning
