# Supplementary material for: Prompt Engineering Paradigms for Medical Applications: Scoping Review
Source: J Med Internet Res. 2024 Sep 10;26:e60501. doi: 10.2196/60501 (PMC11422740; doi:10.2196/60501)
Supplement: Multimedia Appendix 2 [file jmir_v26i1e60501_app2.docx]

## Search strategy

We conducted a comprehensive literature search using Google Scholar, PubMed, Web of Science, Embase, ACL Anthology, ArXiv, MedRxiv, and BioRxiv. 4 searches were performed using the following queries to encompass all three paradigms of prompt engineering, namely PD, PL and PT, in the medical field:

- "Prompt engineering" AND (medical OR clinical OR medicine OR health OR healthcare OR biomedical)
- "Prompt design" AND (medical OR clinical OR medicine OR health OR healthcare OR biomedical)
- ("Prompt based learning" OR "Prompt-based learning" OR "Prompt learning") AND (medical OR clinical OR medicine OR health OR healthcare OR biomedical)
- ("Prompt tuning" OR "Prompt-tuning") AND (medical OR clinical OR medicine OR health OR healthcare OR biomedical)

Paper extraction was conducted using PublishOrPerish [1] to extract Google Scholar results and JabRef [2] for title, abstract, and keyword searches on sources lacking this feature initially (i.e. Google Scholar and ACL). The results of the queries were then saved in Rayyan.ai [3] to select the papers fulfilling the criteria.

## Statistical analysis

Table 2 presents the distribution of screened papers by publication venue, categorizing them based on whether the language of study, English, is explicitly stated, inferred from figures or prompts, or not mentioned. However, the statistical relationship between the type of publication venue and the clarity of how the language of study, English, is disclosed remains ambiguous. To address this, we conducted a Chi-squared test to assess the null hypothesis: "The type of journal is independent of the manner in which the language of study, English, is disclosed, whether stated explicitly, inferred, or not mentioned at all". The found *P*-value is .024, meaning we can reject the null hypothesis. This test was performed on all results based on the distributions with the resulting p-values available in Table S1.

**Table S1.** Results of the chi-squared tests performed.

| **Null hypothesis** | **Involved item** | ***P*-value result** |
| --- | --- | --- |
| The type of journal is independent of the manner in which the language of study, English, is disclosed, whether stated explicitly, inferred, or not mentioned at all | Table 2 | .024 (<.05) |
| The language of study is independent of the manner in which it is disclosed, whether stated explicitly, inferred, or not mentioned at all | Table 2 | <.001 (<.05) |
| The prompt paradigm in the paper is independent of the reported values of the baseline | Table 4a | <.001 (<.05) |
| The type of journal is independent of the reported values of the baseline | Table 4b | <.001 (<.05) |
| The NLP task is independent of the reported values of the baseline | Table 4c | .378 (>.05) |

##

## References

[1] A.-W. Harzing, PublishOrPerish. Url: https://harzing.com/resources/publish-or-perish.

[2] JabRef. Url: <https://www.jabref.org/>

[3] Rayyan. Url: <https://rayyan.ai/>
